# Understanding vaccine hesitancy through the lens of trust and the 3C model: evidence from Chinese General Social Survey 2021

**DOI:** 10.3389/fpubh.2025.1671457

**Published:** 2025-10-01

**Authors:** Bo Dong, Hengxuan Xu, Yuantao Qi, Yifan Li

**Affiliations:** 1 School of Public Health, Zhejiang Chinese Medicine University, Hangzhou, Zhejiang, China; 2 Zhongnan Hospital of Wuhan University, Wuhan, Hubei, China; 3 Shandong Cancer Hospital and Institute, Shandong First Medical University and Shandong Academy of Medical Sciences, Jinan, Shandong, China

**Keywords:** trust, vaccine hesitancy, COVID-19, psychological antecedent, China

## Abstract

**Background:**

Trust is critical in managing infectious disease outbreaks, influencing both healthcare delivery and public compliance. While existing studies suggest trust reduces vaccine hesitancy (VH), the mechanisms remain unclear, particularly how different types of trust impact VH.

**Methods:**

This study uses data from the 2021 Chinese General Social Survey (CGSS), analyzing responses from 7,907 individuals. VH was assessed via COVID-19 vaccination status. Four trust types—generalized, government, doctor, and internet trust—were examined using binary probit regression. Structural equation modeling analyzed the mediating role of psychological factors: self-confidence, complacency, and responsibility. Robustness checks employed alternative dependent variables and models.

**Results:**

Trust exerts a significant negative predictive effect on vaccine hesitancy, suggesting that higher levels reduce the likelihood of vaccine hesitancy. This finding remains statistically significant after robustness tests. However, trust in the government and physician exert a greater influence on vaccine hesitancy than generalized trust and online trust. The three psychological antecedents—confidence, complacency, and collective responsibility—serve a crucial mediating role between trust and vaccine hesitancy. Most vaccinations were community-organized, followed by voluntary and employer-organized vaccination. Higher VH correlated with lower trust across all types, though most hesitancy levels occurred among those with moderate to high trust.

**Conclusion:**

Strengthening trust—especially in government and healthcare providers—is essential to reducing VH. Psychological determinants like confidence, complacency, and responsibility play key roles in vaccination decisions. Tackling VH requires multi-level strategies: fostering public trust, enhancing government transparency, empowering healthcare professionals, combating online misinformation, and leveraging community initiatives.

## 1 Introduction

The COVID-19 pandemic is an unprecedented global crisis caused by infectious diseases for the current generations (1), having profound impacts human health, economic systems, and healthcare infrastructure. According to the latest data from the World Health Organization (WHO), as of December 15, 2024, the global cumulative confirmed COVID-19 cases reached 777,074,803, with 7,079,142 deaths (2). For most known infectious diseases, safe and effective vaccines and successful vaccination programs are essential for controlling disease transmission and reducing morbidity and mortality rates. This is because Vaccination is a safe and effective public health measure that helps protect individuals and social groups from disease (3). During the COVID-19 pandemic, COVID-19 vaccines, as the most important and critical measure to prevent COVID-19 transmission, have played a vital role in the global response to this infectious respiratory disease. Both clinical trials and realistic evidence confirmed the vaccine’s strong protection against infection, severe illness, and death, while maintaining a commendable safety profile (4). Multiple types of COVID-19 vaccines are currently available, including inactivated vaccines, recombinant protein vaccines, adenovirus vector vaccines, attenuated influenza virus vector vaccines, and nucleic acid vaccines (5). These vaccines serve as powerful tools for effectively slowing the spread and transmission of COVID-19.

While countries have embraced increased vaccination as an important public health initiative to protect public health, the protective effects of COVID-19 vaccine have also been demonstrated. However, in many countries, there is still a significant proportion of the population that is hesitant to be vaccinated with COVID-19. This phenomenon poses a major obstacle to the achievement of herd immunity. It not only undermines government efforts to distribute vaccination services but also weakens biomedical companies’ confidence in investing in vaccine technology (6). Globally, achieving higher COVID-19 vaccination rates faces two major challenges: first, addressing the unequal distribution of vaccines among different countries, and second, reducing vaccination barriers and promoting complete vaccination processes. The ultimate goal of vaccination promotion is to transform passive recipients into active seekers, which is particularly important for those who hesitate or refuse to be vaccinated in countries and regions with easy access to vaccination services.

Vaccine hesitancy (VH) refers to a delay in acceptance or refusal of vaccines despite the availability of vaccination services (7). Its spread has accelerated in the last two decades as a result of public distrust in the safety and efficacy of vaccines. The WHO has identified VH as one of the greatest threats to public health at a global level in 2019 (8). In the COVID-19 vaccination service, public concerns about vaccine safety, efficacy, risk, and mistrust are widespread. Data from 23 countries around the world revisited the global hesitancy for the COVID-19 vaccine and found that the average global COVID-19 vaccine rate in 2021 was 24.8% (9). But VH has been observed and reported to vary across populations, countries, and Regions. In the early stages of the COVID-19 outbreak in the United States, an investigation of a representative sample of adults revealed that 57.6% intended to be vaccinated, 31.6% were uncertain, and 10.8% did not intend to receive the vaccination at all (10). A survey conducted in the UK showed that 16.9% of respondents were hesitant to receive COVID-19 vaccines (11), while low rates of COVID-19 vaccine acceptance were reported in the Middle East, Russia, Africa, and several European countries as well (12). A recent survey in Japan found that rural residents were more willing to get vaccinated (70.3%) than those living in central urban areas (62.5%). The results of a survey on attitudes toward COVID-19 vaccination conducted in Thailand found that 90.2% of participants were either willing to be vaccinated or had already been vaccinated, while only 6.0% were hesitant and 3.8% did not want to be vaccinated (13). In China, studies on COVID-19 VH have been conducted mainly in specific locations or among specific groups, with relatively few studies analyzing COVID-19 vaccination and VH using data from large-scale national surveys. The results of a nationwide multicenter online survey showed that the overall hesitancy rate of COVID-19 was about 15%, with students showing the highest rate at about 23%, followed by the general population (21%), doctors (13%), and public health professionals (10%) (14). The results of a cross-sectional study of Chinese people over the age of 18 showed that the VH in the primary vaccination is 8.40%; the VH in booster vaccination is 8.39% (15). Results from the China Health and Retirement Longitudinal Study (CHARLS) showed that as of July/August 2022, 92.3% of China’s population aged 60 years and older had received at least one COVID-19 vaccination, with 88.6% completing the initial shot and 72.4% receiving a booster shot. Vaccination rates among those aged 80 years and older were lower, with 71.9 and 46.7% completing initial and booster vaccinations (16).

The reasons for COVID-19 VH are numerous. Previous studies have confirmed personal characteristics (age, gender, religion, etc.) (17, 18),socioeconomic characteristics (income, education, health insurance, etc.) (19), and the vaccine itself (safety, efficacy, convenience, etc.) all influence VH (20, 21). In addition to the above factors, trust is also an important factor influencing VH, and studies in China and other countries have explored the relationship between trust and COVID-19 VH (22, 23). However, current studies have only analyzed the effect of single types of trust (e.g., government trust, scientific trust, doctor trust, etc.) on VH. There remains a gap in understanding the psychological mechanisms behind trust’s effect on VH, and there is a lack of comparative analysis regarding the differential effects of various types of trust on VH. This lack of understanding of the relationship between trust and VH not only hinders the design of more targeted interventions but also limits the effectiveness of measures to promote vaccination from both the supply and demand sides. Given that COVID-19 epidemics continue to affect public life, lessons should be carefully learned from the practice of COVID-19 vaccination to cope with the uncertainty that new COVID-19 variants may bring and the impact of other infectious diseases in the future.

Therefore, this study takes COVID-19 vaccination as an example. Using questionnaire survey data from mainland Chinese residents, it explores the relationship between vaccine literacy and VH and its potential psychological mechanisms. China plays a crucial role in COVID-19 vaccine coverage, not only because it accounts for one-fifth of the world’s population but also because of its increasingly close connections with other parts of the world. Based on this, the present study explores the relationship between trust and VH and its underlying psychological mechanisms using representative national survey data (Chinese General Social Survey) on COVID-19 vaccination in China as an example. Overall, the innovations of the study are as follows: first, categorizing trust into different types helps to clarify the relationship between different types of trust and VH; second, the study clarifies the mediating pathway between trust and VH. Finally, the study explored the relationship between the level of VH and trust.

## 2 Theoretical framework

Trust is a “psychological state comprising the intention to accept vulnerability based upon the behavior of positive expectations of the intentions of or behavior of another (24). It is an expectation developed and established through social interactions, manifested as belief in someone’s behavior or that the surrounding order aligns with one’s wishes. Previous studies have shown that trust can be largely categorized into social trust and generalized trust. Social trust refers to trust in those “whom people do not personally know or in institutions responsible for regulating or handling certain hazards” (25). Generalized trust is a crucial part of social capital (26), it refers to the differing characteristics between individuals about their willingness to trust other members of society in general (27). This trust is not directed toward specific individuals or groups, but rather represents a broad sense of social trust. Other types of trust—such as trust in government or doctors—are more specific to specific groups or institutions. As the foundation for cooperation among individuals in society, general trust among members fosters higher levels of collaboration (28, 29). General trust helps reduce uncertainty and risk within society, as individuals can depend on others’ goodwill and cooperation in daily life (30, 31). It represents a form of macro-level social capital, typically associated with social welfare, social cohesion, and civic engagement. As infectious diseases pose a threat to society, trust affects people’s risk perception of diseases, which in turn affects vaccination behavior. Numerous studies have discussed the relationship between trust and VH, revealing the effects of social trust and generalized trust on vaccination (32–34). Among social trust, trust in government, media, and doctors are the most important aspects (30, 35). The distinction between these specific trusts and general trusts is presented in Table 1. Previous findings have shown that trust in government and doctors increases vaccine confidence and reduces VH (36–38). Trust in media, however, can have dual effects on vaccination. On the one hand, the popularization of emerging media represented by the Internet has broadened the channels for people to obtain health services and health information, which helps to improve self-health literacy and reduces VH (39). On the other hand, the outbreak of conspiracy theories and undesirable false information on the Internet during epidemics of infectious diseases has also increased people’s concerns about vaccine safety and effectiveness (40). According to the social amplification of risk framework (SARF), media tends to simplify information during transmission, emphasizing risk signals while overlooking other content (41). Therefore, the media is more likely to disseminate simplified information to the general public, which in turn increases risk perception. Studies on COVID-19, SARS, and other infectious diseases have shown that online misinformation can undermine vaccination confidence and increase VH (42, 43). Similar findings are also reflected in the relationship between generalized trust and VH. Liu and Janet (44) demonstrated that social trust negatively correlates with perceived risk and positively correlates with perceived benefits, while also being associated with vaccination willingness. Kunitoki et al. (45) found that increasing social trust can regain vaccination confidence, using HPV vaccination in Japan as an example.

**Table 1:** Analysis of differences among various trust types.

Trust types	Definition	Function	Difference
Generalized trust	Trust in the majority of people or society as a whole	Promote social cooperation, reduce uncertainty, and strengthen social cohesion.	Trust in society as a whole, while other types of trust are more specific, directed toward particular groups or institutions.
Government trust	Individual trust in the government and its policies	Enhance public compliance with government policies and promote the implementation of public health measures.	Focusing on trust in the government and its policies influences public acceptance of public health policies.
Doctor trust	Individuals’ trust in healthcare professionals	Influencing patients’ health decisions, such as whether to receive vaccines or treatment plans	Focusing on individuals’ trust in healthcare professionals directly impacts patients’ health decisions.
Internet trust	Individual trust in online information and internet platforms	The ways in which the public accesses and receives health information determine whether they trust online health information.	Focusing on individuals’ trust in online information sources influences their acceptance of health information found online.

The psychological antecedents can be described as the psychological states or emotions that people hold during the vaccination program. It can reveal the complex psychological mechanisms behind the phenomenon of VH (46). According to the WHO Advisory Group of Experts on Immunization Strategies Working Group’s work and subsequent studies, VH is primarily determined by three psychological factors: confidence, complacency, and convenience prior to vaccination. In 2018, building on the application and theoretical research of the aforementioned model, a German research team expanded it by incorporating two additional dimensions: calculation and collective responsibility (47). In a study designed to assess factors influencing COVID-19 vaccine hesitancy through three psychological factors—confidence, complacency, and convenience—researchers collected data from 7,678 adults aged 18 and older through an online survey. This data were used to analyze the association between VH and related psychological factors. The findings revealed that complacency had the highest explanatory power for VH, accounting for 38% of the variance. Confidence followed, explaining 21% of the variance in VH (48). Furthermore, convenience also emerged as a determinant of VH. In a cross-sectional study conducted in Israel, researchers developed a VH questionnaire based on five core factors—confidence, complacency, convenience, calculation, and collective responsibility—to assess parents’ hesitancy toward COVID-19 vaccination for their children and the factors influencing it. The findings revealed positive correlations between confidence, calculation, and collective responsibility and vaccination willingness, while complacency and calculation showed negative correlations with vaccination willingness (49).

On the other hand, studies have been conducted to analyze the mediating role of vaccination antecedents psychology between other variables and VH. Maietti et al. (50) found that psychological influences mediated the relationship between institutionally sourced information and VH. Guan et al. (51) found that psychological antecedents played a significant mediating role between community involvement and COVID-19 vaccination behavior. Zhou et al. (52) validated that fear of COVID-19, as a component of complacency, would mediate the relationship between social media information and VH. Zhang et al. (53) showed that conspiracy beliefs significantly predicted VH by confidence and complacency of vaccines. All the results suggest that psychological antecedents not only directly influence VH, but also act as mediators to modulate the correlates of VH.

**Figure 1:**
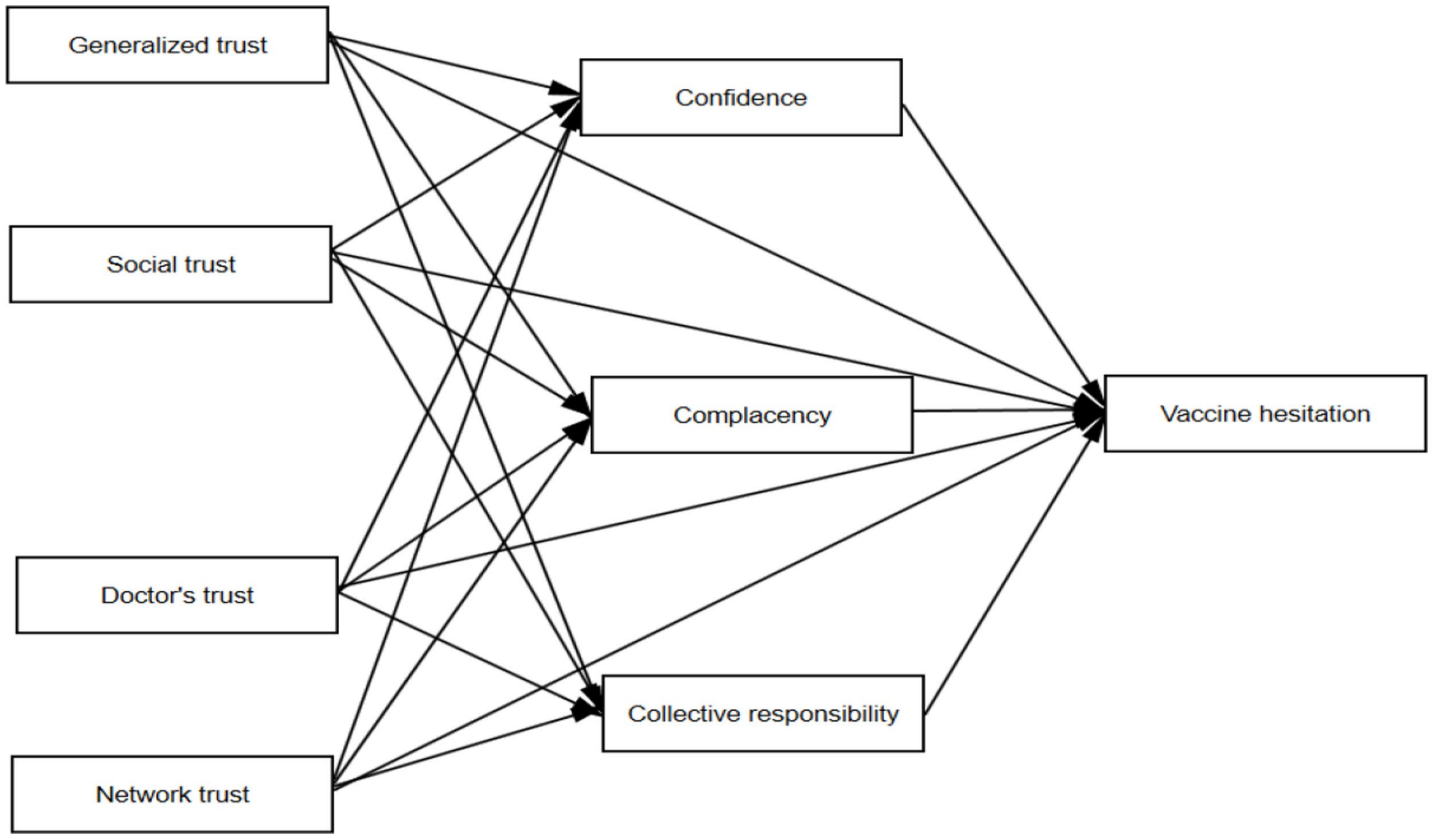
Theoretical framework of the effect of trust on vaccine hesitancy under the psychological mediation of vaccination preamble.

Based on previous literature and theoretical analyses, it was hypothesized that different types of trust are directly and negatively associated with VH, with psychological antecedents of vaccination mediating these relationships. Based on data availability, three psychological antecedents-confidence, complacency, and collective responsibility (3C) – were selected for this study. A theoretical framework was developed to illustrate the pathway and causal hypotheses of the effect of trust on VH and the mediating role of psychological antecedents, as shown in Figure 1.

## 3 Data and methods

### 3.1 Data source

The data used in this study were derived from the 2021 Chinese General Social Survey (CGSS), a large-scale continuous random sample survey project initiated by the China Center for Survey and Data of Renmin University of China in 2003. It represents the earliest national, comprehensive, and continuous academic survey project in China, widely recognized as an authoritative database with an extensive number of survey samples, broad coverage, and diverse content (54).

The survey boasts a comprehensive sample covering 19 provinces, autonomous regions, and municipalities directly under the central government (51), it employs a multi-stage stratified sampling approach, with counties serving as primary sampling units. Post-stratification weights were applied to correct oversampling, ensuring that survey results accurately represented the general population in China (55).

The 2021 CGSS database systematically and comprehensively collects data at multiple levels of society, communities, households, and individuals, such as population demographics, household conditions, labor and employment, social attitudes, and lifestyle. Most importantly, the 2021 survey includes a new section on COVID-19 vaccination status, which documents information on COVID-19 vaccination, pandemic influences on behaviors and attitudes, individual perceptions of vaccination, and many other core factors essential for this study. This rich information, not available in other similar surveys, provides substantial data support for investigating VH.

**Figure 2:**
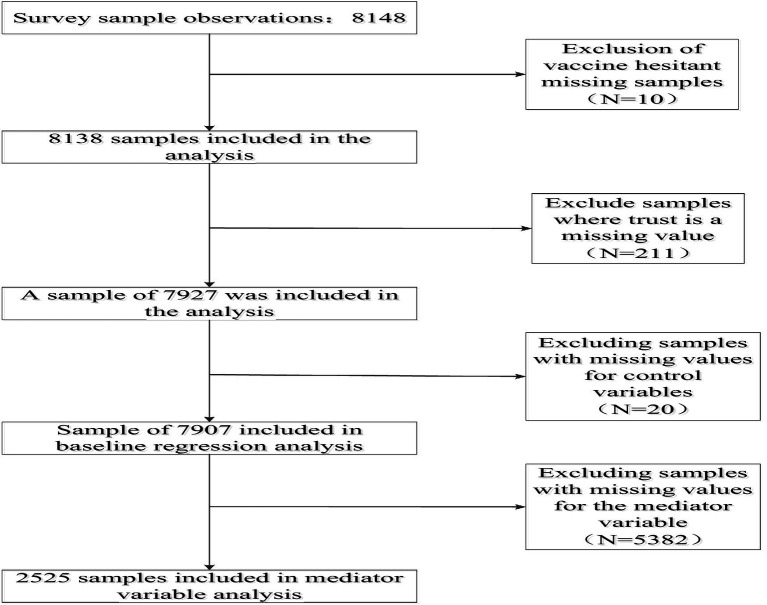
Sample selection process.

The 2021 survey collected a total of 8,148 valid samples nationwide. After processing the samples for missing values, 7,907 samples were retained for this study, and the specific sample selection process can be seen in Figure 2.

### 3.2 Variables

#### 3.2.1 Dependent variable

The dependent variable of interest in this study was VH. In the CGSS2021 questionnaire, the specific question about dependent variables was “Currently, have you been vaccinated against COVID-19?.” Respondents answered with two answers, “vaccinated” and “not vaccinated.” Based on this question, a dummy variable was constructed to measure whether the respondents had been vaccinated against COVID-19. If the answer was “vaccinated,” it was defined that they had been vaccinated against COVID-19 accordingly, otherwise, they had not been vaccinated, i.e., vaccinated = 1; not vaccinated = 0.

#### 3.2.2 Independent variables

Our analysis focuses on four independent variables: generalized trust, government trust, doctor trust, and internet trust. For generalized trust, the study used the question, “In general, do you agree that the vast majority of people in this society can be trusted?” Responses were rated on a 5-point scale from “strongly disagree” (1) to “strongly agree” (5). For government trust, the study utilized the respondents’ confidence in the government. The question posed to the respondents was: “What changes have occurred in your confidence in the government as a result of the measures taken by China to deal with the COVID-19”, and there were five possible choices for the answer, which were “decreased significantly,” “decreased slightly,” “remained unchanged,” “increased slightly,” “increased significantly.” These responses were coded on a scale from 1 to 5, with higher values indicating greater confidence. For doctor trust, this study utilized the following question, “Do you agree that doctors in China are trustworthy,” to which the responses ranged from “strongly disagree” to “strongly disagree.” For Internet trust, the 2021CGSS measured the question, “Do you agree that information on the Internet has had a positive impact on your health behaviors in the past 12 months,” with responses ranging from “Strongly Disagree” to “Strongly Agree” on a scale of 1–5.

#### 3.2.3 Intermediate variables

To explore the mediating pathways through which different types of trust influence VH, this study selected three dimensions—“confidence,” “complacency,” and “collective responsibility”—as mediating variables based on the three psychological antecedents of vaccine hesitancy proposed by the WHO and data availability. The confidence dimension consisted of two questions, i.e., “In general, vaccination has more benefits than drawbacks” and “It is better to gain immunity through vaccination than to get a disease.” Responses to these two questions were measured on a 5-point Likert scale: “strongly disagree,” “disagree,” “neutral,” “agree,” and “strongly agree.” These responses were coded from 1 (strongly disagree) to 5 (strongly agree), and the confidence variable was calculated as the total score for each dimension of the above two questions. The complacency dimension was measured by examining respondents’ primary reasons for not wanting to receive the COVID-19 vaccine. The responses included six options, such as “the epidemic is well controlled, so vaccination is unnecessary” and “concerns about vaccine effectiveness” (see Appendix Table 1 for complete details). The study assigned one point for each selected reason, with scores cumulating up to a maximum of 6 points. The collective responsibility dimension also comprised two questions: “Do you agree that if someone gets infected with COVID-19, it is their own fault?” and “Do you agree that if you get infected with COVID-19, it is your own fault?.” Responses were coded from 1 (strongly disagree) to 5 (strongly agree), and the collective responsibility variable was calculated as the total score for each dimension of the above two questions.

#### 3.2.4 Control variables

In addition to trust factors, an individual’s vaccination behavior may be influenced by other factors. Based on existing related studies (47, 56) and data availability, we control a set of variables in our empirical models to avoid bias from omitted variables. The control variables included in the study specifically include gender, age, income, religion, education, and other 11 factors. The specific definition of each variable can be seen in Appendix Table 2.

### 3.3 Statistical analysis

Descriptive analysis of the demographic characteristics of the participants was performed using frequencies and percentages. The chi-square test was used to analyze whether the differences in vaccination status between different demographic subgroups were statistically significant. Subsequently, to test the relationship between trust and VH, we used employed a binary probit regression model to assess the effect of different types of trust on COVID-19 vaccination behavior.

Structural equation modeling was used to examine the relationship between VH and vaccine literacy, mediated by the “3C” psychological antecedents of vaccination. The bootstrapping method with bias-corrected (BC) confidence intervals (CIs) set at 95% was applied. The total, direct, and indirect effects were estimated using 2000 bootstrapped resamples drawn from the initial sample used for structural equation modeling analysis. The effects are considered to be significant at the *p* ≤ 0.05 when the confidence intervals do not include zero.

In the robustness test section, to investigate the reliability of the findings, this study used the method of replacing the independent variables and replacing the analytical model for robustness analysis. We performed all statistical analyses using STATA version 16.1 (Stata Corp., College Station, TX, United States). Differences were regarded as statistically significant if *p*-values were less than 0.05.

## 4 Results

### 4.1 Basic characterization of respondents

Table 2 shows the descriptive statistics of the main variables in this study. Among the 7,907 respondents, 45.57% were male and 54.43% were female. The proportion of older adults aged 60 years and above was 34.63%. The education level of the respondents was generally low, with only 21.07% having university-level education or above, while 78.93% had high school education or below Regarding household registration status, 66.88% were registered as rural residents and 32.12% as urban residents. Most respondents reported good health status, with 54.09% in good health and 27.98% in fair health. Regarding religious affiliation, 92.59% reported no religious beliefs. The regional distribution of respondents showed that 57.43% were from eastern regions, while 24.70 and 17.87% were from central and western regions, respectively.

**Table 2:** Analysis of the basic characteristics of the respondents.

Variables		*N*	%
Gender	Male	3,603	45.57
	Female	4,304	54.43
Age	≤29	1,163	14.70
	30–59	4,007	50.68
	≥60	2,738	34.63
Marriage	Unmarried	2,279	28.82
	Married	5,628	71.18
Ethnicity	Han Ethnicity	7,330	92.70
	Ethnic Minority	577	7.30
Income	<20,000	1,222	15.45
	20,000-	1,376	17.40
	50,000-	1,477	19.94
	100,000-	1,590	20.11
	200,000-	2,142	27.09
Religious Belief	No Religious Affiliation	7,231	92.59
	Religious Affiliation	586	7.41
Education	Below High School	4,772	60.35
	High School	1,469	18.58
	University and Above	1,666	21.07
work	Unemployed	3,902	49.35
	Employed	4,005	50.65
Health	Unhealthy	1,418	17.93
	Basically Healthy	2,212	27.98
	Healthy	4,277	54.09
Household Registration	Rural	5,288	66.88
	Urban	2,619	32.12
Migration Status	Non-migrant	5,586	70.65
	Migrant Population	2,321	29.35
Region	Western	1,413	17.87
	Central	1,359	24.70
	Eastern	4,541	57.43

### 4.2 Analysis of VH differences among respondents in the baseline survey

Univariate analyses were conducted using vaccination status as the dependent variable, with results presented in Table 3. The differences in VH between different genders, ages, education levels, marriages, jobs, household registration, incomes, religions, work statuses, health, and regions were statistically significant (all *p* < 0.05), while the differences in VH of the respondents between the different mobility statuses were not statistically significant.

**Table 3:** Analysis of differences in vaccine hesitancy among respondents in the baseline survey.

Variables		Unvaccinated		Vaccinated		*χ* ^2^	*p*
		*n*	%	*n*	%		
Gender	Male	993	48.11	2,610	44.67	7.284	0.007
	Female	1,071	51.09	3,233	55.33		
Age	≤29	179	8.67	983	16.82	769.454	0.000
	30–59	655	31.73	3,352	57.37		
	≥60	1,230	59.59	1,508	25.81		
Marriage	Unmarried	637	30.86	1,642	28.10	5.665	0.017
	Married	1,427	69.14	4,201	71.90		
Ethnicity	Han Ethnicity	1951	94.53	5,379	92.06	13.714	0.000
	Ethnic Minority	113	5.47	464	7.94		
Income	<20,000	417	20.20	805	13.78	54.420	0.000
	20,000-	318	15.41	1,058	18.11		
	50,000-	395	19.14	1,182	20.23		
	100,000-	371	17.97	1,219	20.86		
	200,000-	563	27.28	1,579	27.02		
Religious belief	No religious Affiliation	1884	91.28	5,437	93.05	6.983	0.008
	Religious Affiliation	180	8.72	404	6.95		
Education	Below High School	1,414	68.51	3,358	57.47	117.457	0.000
	High School	382	18.51	1,087	18.60		
	University and Above	268	12.98	1,398	23.93		
Work	Unemployed	1,361	65.94	2,541	43.49	307.594	0.000
	Employed	703	34.06	3,302	56.51		
Health	Unhealthy	619	29.99	799	13.67	320.025	0.000
	Basically Healthy	604	29.26	1,608	27.52		
	Healthy	841	40.75	3,436	58.18		
Household registration	Rural	1,464	70.93	3,824	65.45	20.710	0.000
	Urban	600	29.07	2019	34.55		
Region	Western	307	14.87	1,106	18.93	78.697	0.000
	Central	401	19.43	1,552	26.56		
	Eastern	1,356	65.70	3,182	54.51		

### 4.3 The effect of trust on VH

Statistically significant variables from the univariate analysis were used as independent variables, with respondents’ VH as the dependent variable, to conduct probit regression analysis. The results are presented in Tables 4, 5. Table 4 presents the crude model analysis (MODEL 1) without control variables, while Table 5 presents the adjusted model (MODEL 2) incorporating control variables. The regression results and average marginal effect estimates from both MODEL 1 and MODEL 2 indicate that generalized trust, trust in the government, trust in doctors, and trust in online information negatively influence VH among respondents. These effects are statistically significant at the 1% level, indicating that higher levels of trust significantly reduce the probability of VH.

**Table 4:** Effect of trust on vaccine hesitancy (MODEL 1).

Variables	MODEL 1							
	Vaccine hesitancy							
	Regression coefficient	Average processing effect	Regression coefficient	Average processing effect	Regression coefficient	Average processing effect	Regression coefficient	Average processing effect
Generalized trust	−0.054*** (0.014)	−0.017*** (0.005)						
Government trust			−0.135*** (0.018)	−0.044*** (0.006)				
Doctor trust					−0.081*** (0.017)	−0.026*** (0.005)		
Internet trust							−0.066*** (0.020)	−0.021*** (0.006)
*R* ^2^	0.002		0.006		0.003		0.001	
*N*	7,907							

Standard errors are shown in parentheses *** denote significance levels of 1%.

**Table 5:** Effect of trust on vaccine hesitancy (MODEL 2).

Variables	MODEL 2							
	Vaccine hesitancy							
	Regression coefficient	Average processing effect	Regression coefficient	Average processing effect	Regression coefficient	Average processing effect	Regression coefficient	Average processing effect
Generalized trust	−0.093*** (0.016)	−0.027*** (0.004)						
Government trust			−0.256*** (0.019)	−0.072*** (0.005)				
Doctor trust					−0.197*** (0.018)	−0.056*** (0.005)		
Internet trust							−0.154*** (0.021)	−0.044*** (0.066)
Gender	0.168*** (0.033)	0.064*** (0.009)	0.228*** (0.034)	0.061*** (0.009)	0.215*** (0.034)	0.051*** (0.010)	0.178*** (0.033)	0.048*** (0.009)
Age	−0.472*** (0.029)	−0.147*** (0.008)	−0.518*** (0.029)	−0.146*** (0.008)	−0.512*** (0.029)	−0.139*** (0.008)	−0.483*** (0.029)	−0.136*** (0.008)
Marriage	0.189*** (0.037)	0.049*** (0.011)	0.172*** (0.037)	0.051*** (0.010)	0.181*** (0.037)	0.053*** (0.010)	0.186*** (0.038)	0.054*** (0.010)
Ethnicity	0.090 (0.068)	0.026 (0.020)	0.134* (0.069)	0.038* (0.019)	0.113* (0.068)	0.032* (0.019)	0.102 (0.068)	0.029 (0.020)
Income	−0.022* (0.012)	−0.006* (0.004)	−0.020* (0.012)	−0.006* (0.003)	−0.022* (0.012)	−0.006* (0.004)	−0.020 (0.012)	−0.006 (0.004)
Religious Belief	−0.112* (0.061)	−0.032* (0.018)	−0.099 (0.062)	−0.028 (0.017)	−0.104* (0.061)	−0.030* (0.017)	−0.102* (0.061)	−0.029* (0.018)
Education	0.048* (0.025)	0.015** (0.007)	0.053** (0.025)	0.015** (0.007)	0.052** (0.024)	0.013* (0.007)	0.045* (0.025)	0.014* (0.007)
Work	0.324*** (0.035)	0.097*** (0.010)	0.342*** (0.035)	0.097*** (0.010)	0.339*** (0.035)	0.096*** (0.010)	0.334*** (0.035)	0.093*** (0.006)
Health	0.222*** (0.022)	0.064*** (0.006)	0.228*** (0.022)	0.064*** (0.006)	0.226*** (0.022)	0.064*** (0.006)	0.223*** (0.021)	0.064*** (0.006)
Household registration	0.233*** (0.037)	0.058*** (0.011)	0.206*** (0.038)	0.060*** (0.011)	0.209*** (0.037)	0.064*** (0.011)	0.224*** (0.038)	0.067*** (0.011)
Region	−0.163*** (0.021)	−0.053*** (0.006)	−0.189*** (0.022)	−0.052*** (0.006)	−0.182*** (0.022)	−0.049*** (0.006)	−0.170*** (0.022)	−0.047*** (0.006)
*R* ^2^	0.114		0.130		0.124		0.116	
*N*	7,907							

Standard errors are shown in parentheses; *, **, and *** denote significance levels of 10, 5, and 1%, respectively.

### 4.4 Mediation analysis of the effect of trust on VH

#### 4.4.1 Model fit coefficients

In this study, various types of trust were used as independent variables, self-confidence, complacency, and collective responsibility as mediating variables, and VH as dependent variables. AMOS 25.0 was utilized to set up the basic mediator model for single-step multiple, and the fitness test was conducted to correct the basic model based on the fit results. Table 6 shows the model fit indices in which the *χ*^2^ of fitness is 25.922, corresponding to a *p*-value greater than 0.05, indicating good fit between the modified structural equation model and the sample data. In addition, the RMSEA value is 0.031, the CFI is greater than 0. 90, the GFI is greater than 0. 90, and the AGFI is greater than 0. 90, which indicates that the relevant indexes are within a reasonably acceptable range. These results suggest good overall model fit, supporting the use of this model for path analysis.

**Table 6:** Model fit index.

Evaluation indicators	Model results	Adaptation standards	Fitness judgment
*χ*^2^/df	8.641	<10.00	Yes
RMSEA	0.031	< 0.08	Yes
CFI	0.972	>0.90	Yes
GFI	0.999	>0.90	Yes
AGFI	0.999	>0.90	Yes
NFI	0.969	>0.90	Yes

#### 4.4.2 Logical structure between trust and VH

**Figure 3:**
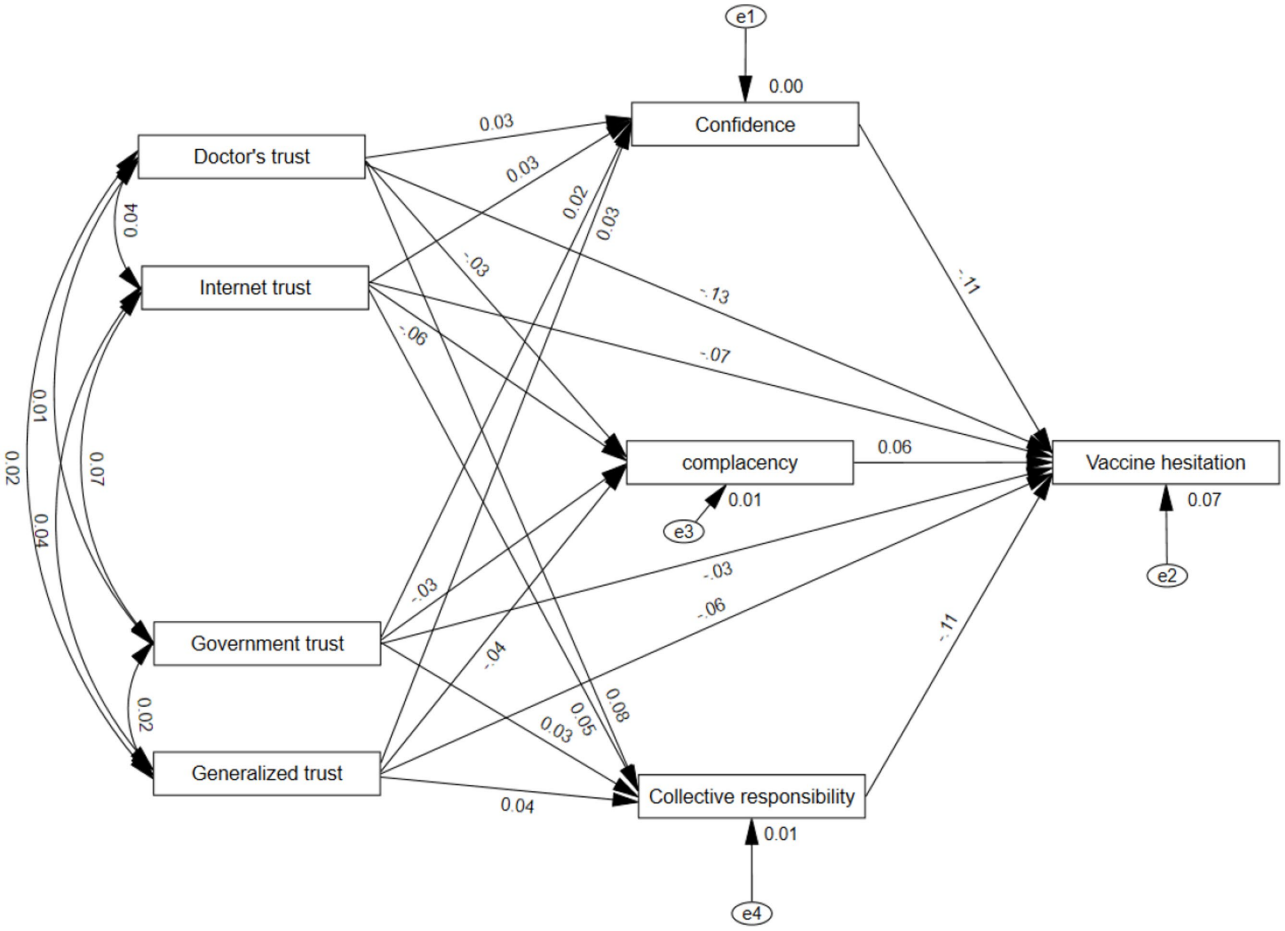
Mediating pathways of trust affecting vaccine hesitancy.

Next is the estimation of the coefficients of the mediated paths. In this study, Bootstrap method is used to set up 5,000 repeated random samples and 95% confidence intervals. The standardized regression coefficients served as evaluation criteria for unidirectional path estimation. Due to AMOS25’s limitation in displaying significance levels for standardized results, unstandardized significance levels indicate overall significance. Figure 3 demonstrates the path of action of various types of trust in influencing VH, while Table 7 specifically presents the regression results with self-confidence, complacency, and collective responsibility as mediators. These results show that generalized trust has a significant negative direct effect on respondents’ VH, with a coefficient size of −0.064. Generalized trust demonstrates a significant positive impact on self-confidence, with a coefficient size of 0.029 at the 5% level of test; a significant negative effect on complacency, with a coefficient size of −0.038 at the 1% level of test; a significant positive impact on collective responsibility, with a coefficient size of 0.043 at the 1% level of test. Both self-confidence and collective responsibility have a significant negative effect on VH at the 1% test level, with the size of the coefficient being −0.109 and −0.111, respectively; and complacency has a significant positive effect on VH at the 1% test level, with the size of the coefficient being 0.064. These findings suggest indirect effect of generalized trust on VH from self-confidence, complacency, and collective responsibility.

**Table 7:** Results of mediated path test for trust affecting vaccine hesitancy.

Path	Non-standardized coefficient	Standardized coefficient	SE	CR	*P*
Generalized trust → Vaccine hesitancy	−0.028	−0.064	0.005	−5.869	<0.001
Generalized trust → Confidence	0.016	0.029	0.006	2.582	<0.05
Confidence → Vaccine hesitancy	−0.084	−0.109	0.008	−10.039	<0.001
Generalized trust → Complacent	−0.024	−0.038	0.007	−3.455	<0.001
Complacent → Vaccine hesitancy	0.044	0.064	0.007	5.935	<0.001
Generalized trust → Collective responsibility	0.033	0.043	0.009	3.86	<0.001
Collective responsibility → Vaccine hesitancy	−0.063	−0.111	0.006	−10.214	<0.001
Government trust → Vaccine hesitancy	−0.019	−0.03	0.007	−2.811	<0.05
Government trust → Confidence	0.019	0.024	0.009	2.106	<0.05
Government trust → Complacent	−0.025	−0.028	0.01	−2.519	<0.05
Government trust → Collective responsibility	0.038	0.034	0.012	3.1	<0.05
Doctor trust → Vaccine hesitancy	−0.109	−0.128	0.009	−11.777	<0.001
Doctor trust → Confidence	0.035	0.032	0.012	2.906	<0.05
Doctor trust → Complacent	−0.036	−0.029	0.014	−2.593	<0.05
Doctor trust → Collective responsibility	0.112	0.075	0.016	6.78	<0.001
Internet trust → Vaccine hesitancy	−0.046	−0.074	0.007	−6.801	<0.001
Internet trust → Confidence	0.022	0.027	0.009	2.448	<0.05
Internet trust → Complacent	−0.054	−0.061	0.01	−5.449	<0.001
Internet trust → Collective responsibility	0.053	0.05	0.012	4.452	<0.001

Government trust has a significant negative direct effect on respondents’ VH with a coefficient size of −0.030, while it has a significant positive effect on self-confidence with a coefficient size of 0.024 at the 5% test level, on complacency with a significant positive effect at the 5% test level with a coefficient size of −0.028, and collective responsibility with a significant positive effect at the 5% test level with a coefficient size of 0.034. At the 1% significance level, both self-confidence and collective responsibility demonstrate significant negative effects on VH, while complacency shows a significant positive effect. These findings indicate that government trust has indirect effects on VH through the mediating roles of self-confidence, complacency, and collective responsibility.

Doctor trust has a significant negative direct effect on respondents’ VH, with a coefficient size of −0.128. It has a significant positive effect on self-confidence at the 5% test level, with a coefficient size of 0.032; a significant negative effect on complacency at the 5% test level, with a coefficient size of −0.029; a significant positive effect on collective responsibility at the 1% test level, with a coefficient size of 0.075. Both self-confidence and collective responsibility have a significant negative effect on VH at the 1% test level; complacency has a significant positive effect on VH at the 1% test level. These results indicate that there is an indirect effect of doctor trust on VH from self-confidence, complacency and collective responsibility.

Network trust has a significant negative direct effect on respondents’ VH, with a coefficient size of −0.074. It has a significant positive effect on self-confidence at the 5% test level, with a coefficient size of 0.105; complacency has a significant negative effect on complacency at the 1% test level, with a coefficient size of −0.061; and collective responsibility has a significant positive effect on collective responsibility at the 1% test level, with a coefficient size of 0.05; and self-confidence, complacency and collective responsibility have a significant effect on VH at the 1% test level, which in turn indicates that there is an indirect effect of network trust on VH from self-confidence, complacency and collective responsibility.

#### 4.4.3 Results of a mediation effect test of trust affecting VH

To analyze the mechanism of trust in influencing VH, this study further decomposes its specific pathways, with the pathway decomposition results presented in Table 8. It can be seen that, in the relationship between generalized trust and VH, the indirect effect of self-confidence accounts for 2.450% of the total effect, the indirect effect of complacency accounts for 1.320% of the total effect, and the indirect effect of collective responsibility accounts for 5.880%. In the relationship between government trust and VH, the indirect effect of self-confidence was 6.650% of the total effect, the indirect effect of complacency was 4.583% of the total effect, and the indirect effect of collective responsibility was 9.975% of the total effect. In the relationship between doctor trust and VH, the indirect effect of self-confidence was 4.200% of the total effect in the relationship between network trust and VH, the indirect effect of self-confidence was 3.300% of the total effect, and the indirect effect of collective responsibility was 6.497% of the total effect. In the relationship between network trust and VH, the indirect effect of self-confidence was 3.487% of the total effect, the indirect effect of complacency was 4.483% of the total effect, and the indirect effect of collective responsibility was 6.300%. The 95% CIs of the total, direct, and indirect effects of the three mediating variables in different items did not contain 0, indicating that the mediating effects were significant, i.e., self-confidence, complacency, and collective responsibility mediated between different types of trust and VH.

**Table 8:** Results of the decomposition of the mediating effect of trust on vaccine hesitancy.

Path	Total effect	Direct effect	Mediation effect					
			Variables	Effect	Effect Proportion	95% BOOT		Effect type
						Lower limit	Upper limit	
Generalized trust → Vaccine hesitancy	−0.032	−0.028	Confidence	−0.001	2.450%	−0.003	0.000	Partial Mediation
			Complacent	−0.001	1.320%	−0.002	0.000	Partial
			Collective responsibility	−0.002	5.880%	−0.003	−0.001	Mediation
Government trust → Vaccine hesitancy	−0.024	−0.019	Confidence	−0.002	6.650%	−0.003	0.000	Partial
			Complacent	−0.001	4.583%	−0.002	0.000	Mediation
			Collective responsibility	−0.002	9.975%	−0.004	−0.001	Partial
Doctor trust → Vaccine hesitancy	−0.120	−0.109	Confidence	−0.003	4.200%	−0.005	−0.001	Mediation
			Complacent	−0.002	3.300%	−0.003	0.000	Partial
			Collective responsibility	−0.007	6.497%	−0.010	−0.005	Mediation
Internet trust → Vaccine hesitancy	−0.053	−0.046	Confidence	−0.002	3.487%	−0.004	0.000	Partial
			Complacent	−0.002	4.483%	−0.004	−0.001	Mediation
			Collective responsibility	−0.003	6.300%	−0.005	−0.002	Partial

**Figure 4:**
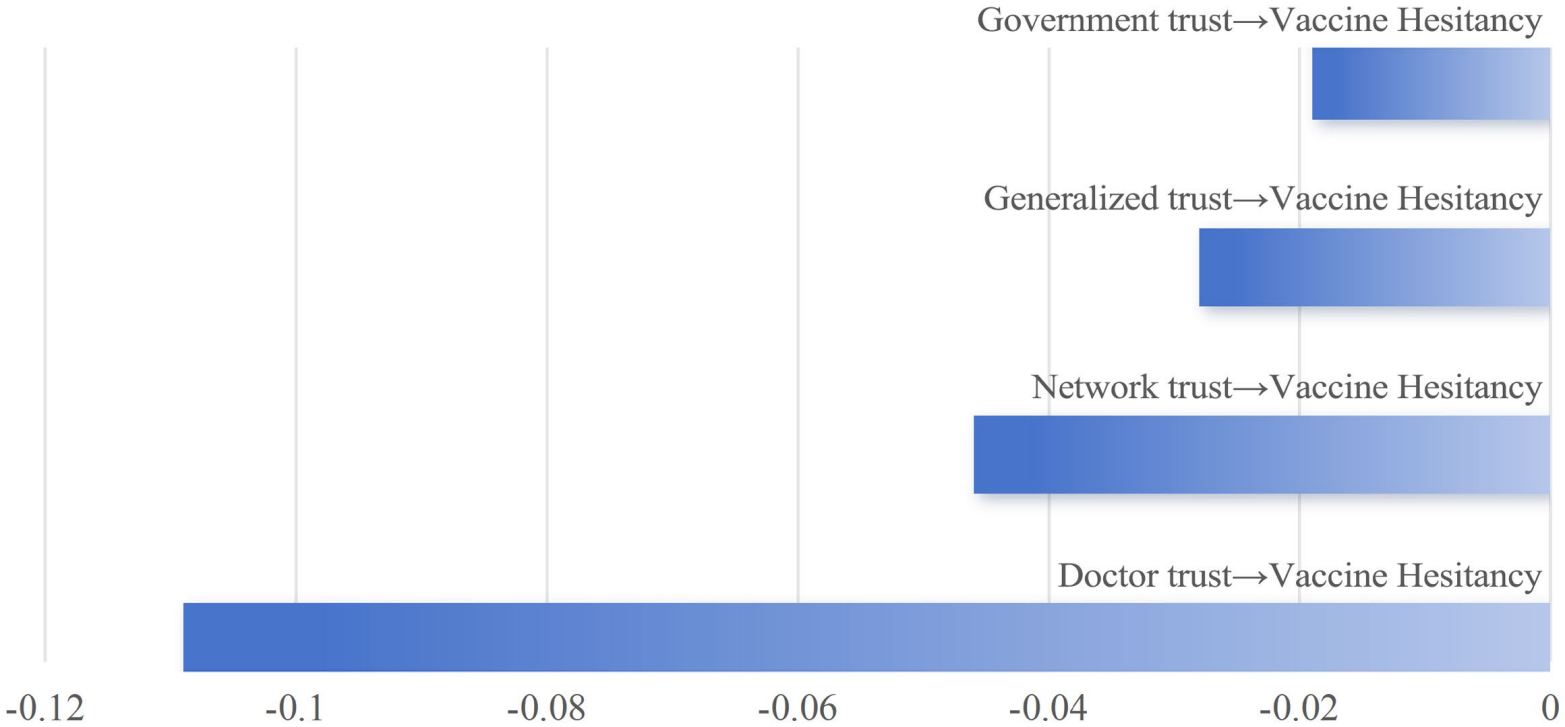
Direct effects of different trust types on vaccine hesitancy.

**Figure 5:**
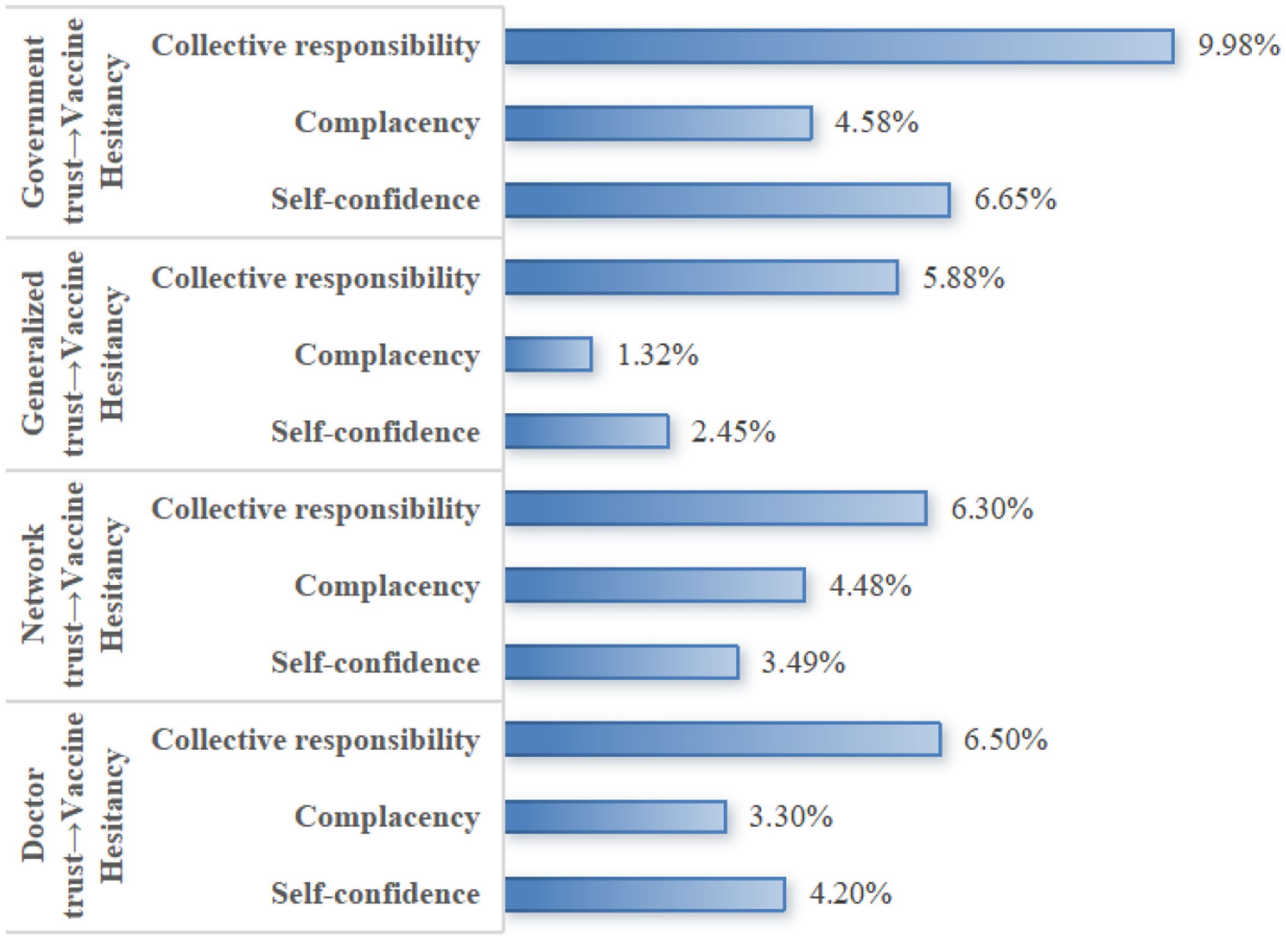
Comparing the indirect effects of different trust types on vaccine hesitancy.

The comprehensive analysis reveals that different types of trust exert significant negative direct effects on VH. The impact strength, from highest to lowest, is as follows: trust in doctors, trust in online sources, generalized trust, and trust in the government (Figure 4). Furthermore, different trust types exert indirect effects on VH through distinct psychological mechanisms. The indirect effect of collective responsibility accounts for a substantial proportion in most trust types, particularly in government trust, physician trust, and online trust, where it exhibits the highest proportion (Figure 5).

### 4.5 Relationship between trust and levels of VH

Regarding respondents’ vaccination methods, this study used the question “Was your COVID-19 vaccination organized by someone?” from the 2021 CGSS questionnaire. The results indicate that community notification or organization was the primary vaccination method (57.64%), followed by self-initiated vaccination (23.66%), while workplace notification or organization accounted for the lowest proportion (18.70%).

**Figure 6:**
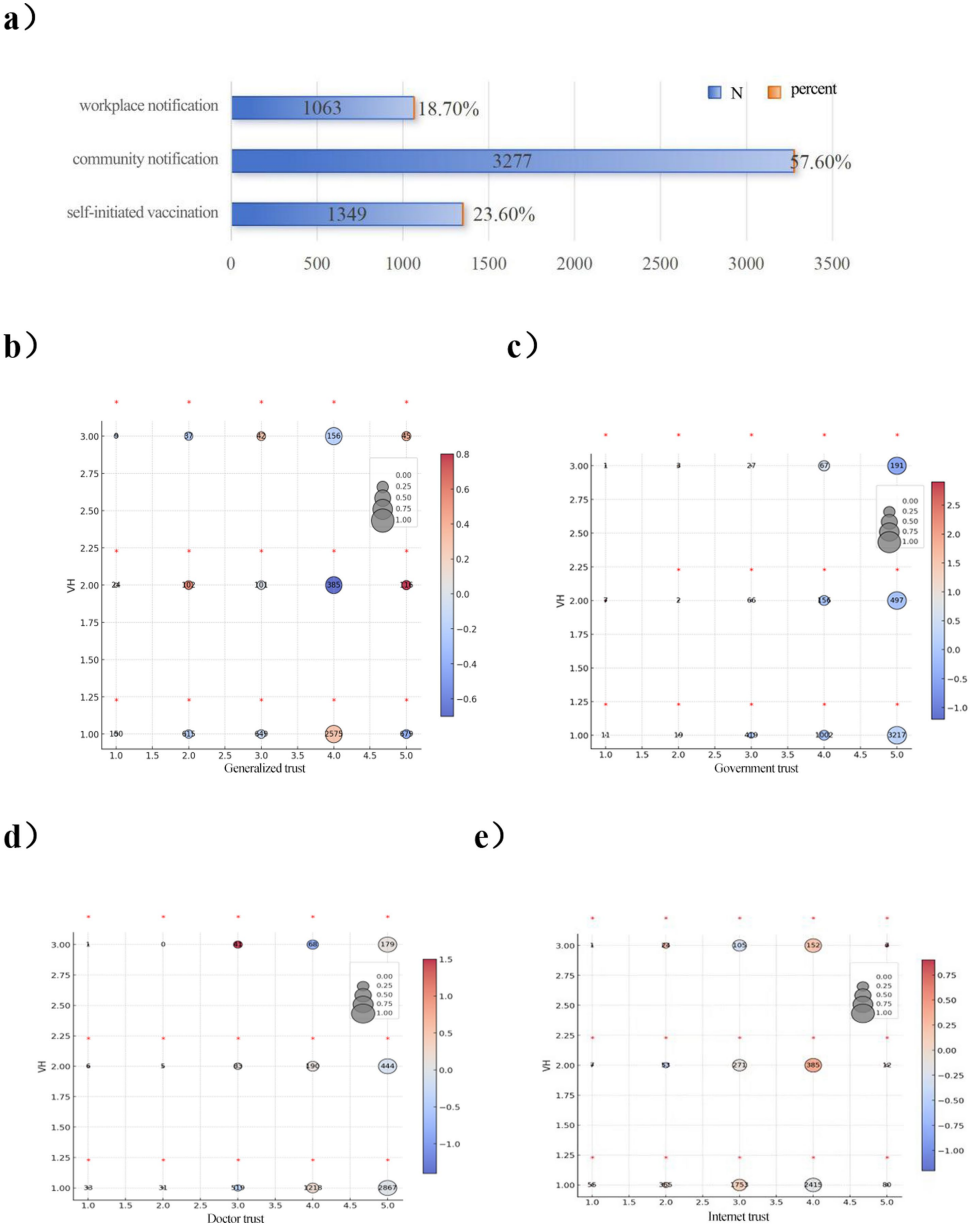
Level of vaccine hesitation and type of trust. This figure shows a bar graph and 5 contingency tables that follow the scale located in the upper right section of the figure. **(a)** Respondents’ approach to COVID-19 vaccines. **(b)** Distribution of vaccine hesitancy on Generalized trust. **(c)** Distribution of vaccine hesitancy on government trust. **(d)** Distribution of vaccine hesitancy on doctor trust. **(e)** Distribution of vaccine hesitancy on internet trust. The numbers inside the circles show the absolute frequency, the size of the circle indicates the proportion relative to the level of VH, and the color of the circle shows the value of the standardized residuals of the Chi-squared for each cell. The standardized residuals whose value is higher than 2 or lower than −2 are considered as significant differences and are highlighted with an asterisk above the circle.

Meanwhile, to analyze the relationship between social trust and VH levels, this study used the question “Which of the following statements best describes your situation regarding COVID-19 vaccination?” from the 2021 CGSS questionnaire. This question was used to measure the level of VH, and there were three responses to this question, “I did not want to get vaccinated at al from the beginning,” “I was hesitant to get vaccinated at first, but I finally got vaccinated,” and “I did not want to get vaccinated at first, but I finally got vaccinated.” According to the degree of hesitation, these three responses were assigned a value of 1, 2, and 3, respectively. It can be seen that the degree of VH is significantly correlated with generalized trust, government trust, doctor trust, and network trust. The overall trend shows that higher levels of VH correspond to lower levels of trust across all dimensions. Despite significant variations in VH levels, respondents generally maintained medium to high trust levels across all trust categories. In the generalized trust dimension, more than 80% of respondents in each VH group scored 3 or higher on the generalized trust level; in the government trust dimension, more than 89% of respondents in each VH group scored 3 or higher on the generalized trust level; in the doctor trust dimension, more than 95% of respondents in each VH group scored 3 or higher on the generalized trust level; and in the Internet trust dimension, the more than 90% of respondents in each VH group scored the level of generalized trust as 3 or higher (Figure 6).

### 4.6 Robustness test

#### 4.6.1 Replacement of the dependent variable

To validate the robustness of the analytical results, this study first employed a robustness test by replacing the dependent variable. In this robustness test, different levels of VH were used as a proxy variables for hesitancy. An ordered Probit regression model was applied to analyze the relationship between various types of trust and hesitancy, with results presented in Tables 9, 10. Table 9 presents the crude model analysis (MODEL 3) without control variables, while Table 10 presents the results after adjusting for control variables (MODEL 4). The regression results and average marginal effect estimates in both MODEL 3 and MODEL 4 indicate that trust significantly and negatively predicts VH, regardless of whether control variables are included. This confirms the robustness of the study’s findings.

**Table 9:** Robustness tests for substituting dependent variables (MODEL 3).

Variables	MODEL 3							
	Vaccine hesitancy							
	Regression coefficient	Average processing effect	Regression coefficient	Average processing effect	Regression coefficient	Average processing effect	Regression coefficient	Average processing effect
Generalized trust	−0.007** (0.003)	−0.042*** (0.004)						
Government trust			−0.273*** (0.024)	−0.042*** (0.004)				
Doctor trust					−0.084*** (0.023)	−0.013*** (0.004)		
Internet trust							−0.071** (0.026)	−0.011*** (0.004)
*R* ^2^	0.001		0.019		0.002		0.001	
N	5,685							

Standard errors are shown in parentheses; **, *** denote significance levels of 5, 1%, respectively.

**Table 10:** Robustness tests for substituting dependent variables (MODEL 4).

Variables	MODEL 4							
	Vaccine hesitancy							
	Regression coefficient	Average processing effect	Regression coefficient	Average processing effect	Regression coefficient	Average processing effect	Regression coefficient	Average processing effect
Generalized trust	−0.048** (0.019)	−0.005*** (0.002)						
Government trust			−0.289*** (0.024)	−0.029*** (0.003)				
Doctor trust					−0.090*** (0.023)	−0.009*** (0.002)		
Internet trust							−0.074** (0.026)	−0.008*** (0.003)
Gender	0.003 (0.039)	0.000 (0.004)	0.004 (0.039)	0.000 (0.004)	0.006 (0.039)	0.001 (0.004)	0.007 (0.039)	0.001 (0.004)
Age	0.014 (0.035)	0.001 (0.004)	0.029** (0.035)	0.003 (0.004)	0.017 (0.035)	0.002 (0.004)	0.018 (0.035)	0.002 (0.004)
Marriage	0.049 (0.044)	0.005 (0.005)	0.126** (0.046)	0.013*** (0.005)	0.045 (0.044)	0.005 (0.005)	0.043 (0.044)	0.004 (0.005)
Ethnicity	0.047 (0.073)	0.005 (0.008)	0.087 (0.074)	0.009 (0.008)	0.047 (0.073)	0.005 (0.008)	0.034 (0.073)	0.004 (0.008)
Income	−0.009 (0.015)	−0.001 (0.002)	−0.004 (0.015)	−0.000 (0.002)	−0.006 (0.015)	−0.001 (0.002)	−0.006 (0.015)	−0.001 (0.002)
Religious belief	−0.051 (0.077)	−0.005 (0.008)	−0.047 (0.077)	−0.005 (0.008)	−0.051 (0.076)	−0.005 (0.008)	−0.047 (0.076)	−0.005 (0.008)
Education	0.023 (0.029)	0.002 (0.003)	0.041 (0.029)	0.004 (0.003)	0.028 (0.029)	0.003 (0.003)	0.021 (0.029)	0.002 (0.003)
Work	0.089** (0.041)	0.009** (0.004)	0.084** (0.041)	0.009** (0.004)	0.100** (0.041)	0.010** (0.004)	0.100 (0.041)	0.010** (0.004)
Health	−0.015 (0.027)	−0.002 (0.003)	0.002 (0.027)	0.000 (0.003)	−0.008 (0.027)	−0.001 (0.003)	−0.011 (0.027)	−0.001 (0.003)
Household registration	−0.051 (0.044)	−0.005 (0.005)	−0.059 (0.044)	−0.006 (0.005)	−0.053 (0.044)	−0.006 (0.005)	−0.049 (0.044)	−0.005 (0.005)
Region	−0.015 (0.025)	−0.002 (0.003)	−0.016 (0.025)	−0.002 (0.003)	−0.015 (0.025)	−0.002 (0.003)	−0.015 (0.025)	−0.002 (0.003)
*R* ^2^	0.003		0.022		0.004		0.003	
*N*	5,685							

Standard errors are shown in parentheses; **, *** denote significance levels of 5, 1%, respectively.

#### 4.6.2 Replacement analysis model

In the robustness tests, this study further employed a model substitution approach to validate the findings by replacing the binary Probit model with a binary logistic model to estimate the impact of trust on VH (Table 11). The results indicate that trust also exerts a significant negative effect on respondents’ VH. These empirical findings align with the earlier conclusions, further confirming the robustness of the research results.

**Table 11:** Robustness test of the substitution analysis model.

Variables	*β*	95%CI	*P*	*β*	95%CI	*P*	*β*	95%CI	*P*	*β*	95%CI	*P*
Generalized trust	−0.403	−0.484–−0.320	0.000									
Government trust				−0.686	−0.823–−0.548	0.000						
Doctor trust							−0.712	−0.836–−0.587	0.000			
Internet trust										−0.524	−0.638–−0.411	0.000
Gender	0.215	0.071–0.358	0.003	0.197	0.053–0.340	0.007	0.199	0.056–0.343	0.006	0.222	0.079–0.364	0.002
Age	−1.146	−1.281–−1.011	0.000	−1.1192	−1.2542–−0.984	0.000	−1.134	−1.269–−0.999	0.000	−1.166	−1.301–−1.031	0.000
Marriage	0.337	0.181–0.493	0.000	0.2971	0.140–0.454	0.000	0.304	0.147–0.461	0.000	0.329	0.174–0.484	0.000
Ethnicity	0.250	−0.066–0.566	0.121	0.3924	0.074–0.710	0.016	0.369	0.053–0.689	0.022	0.296	−0.021–0.609	0.068
Income	−0.048	−0.098–0.003	0.067	−0.0425	−0.093–0.009	0.102	−0.049	−0.100–0.002	0.059	−0.045	−0.095–0.006	0.083
Religious Belief	−0.403	−0.650–−0.157	0.001	−0.3847	−0.631–−0.139	0.002	−0.379	−0.626–−0.131	0.003	−0.371	−0.617–−0.125	0.003
Education	0.146	0.032–0.260	0.012	0.1802	0.066–0.294	0.002	0.183	0.069–0.297	0.002	0.153	0.040–0.266	0.008
Work	0.679	0.519–0.837	0.000	0.6556	0.497–0.814	0.000	0.674	0.516–0.832	0.000	0.682	0.534–0.849	0.000
Health	0.518	0.427–0.609	0.000	0.5440	0.497–0.814	0.000	0.542	0.450–0.633	0.000	0.525	0.434–0.615	0.000
Household Registration	0.517	0.353–0.682	0.000	0.4664	0.303–0.630	0.000	0.471	0.307–0.635	0.000	0.498	0.336–0.660	0.000
Region	−0.315	−0.411–−0.218	0.000	−0.3038	−0.401–−0.207	0.000	−0.305	−0.402–−0.208	0.000	−0.311	−0.407–−0.215	0.000
*N*	7,907											
*R* ^2^	0.191			0.193			0.199			0.189		

## 5 Discussion

Given China’s vast population and its substantial contributions to global pandemic control efforts, this study uses nationally representative survey data collected prior to the implementation of mainland China’s zero-COVID policy to confirm a significant negative association between trust and VH. Moreover, the “3C” psychological antecedents of vaccination critically mediate this relationship. Additionally, the quantitative relationship between trust and VH levels has been established. These findings provide further evidence supporting strategies to reduce VH by addressing factors related to trust.

Our findings suggest that generalized trust, government trust, doctor trust, and internet trust all have a significant effect on VH and that all four dimensions of trust contribute to the reduction of VH. This is consistent with previous studies showing that trust, as an important social factor influencing vaccination, is an important factor in reducing VH. For example, a study using data from the 2018 Wellcome Global Monitor survey (comprising nationally representative samples from 144 countries; *n* = 149,014 respondents) analyzed the correlation between trust levels in government and health workers and attitudes toward vaccines. Globally, only one-quarter of respondents highly trusted governments, and less than half of respondents highly trusted doctors and nurses. Trust in these institutions was correlated with trust in their health or medical advice and more positive attitudes toward vaccines (57). The results of an analysis using macro- and micro-level data from China suggest that trust in local government and the media contributes to lower rates of COVID-19 disease infection. The effects of different types of trust are moderated by perceptions of disease risk (58).

Unlike previous studies, this research further analyzes the relationship between different types of trust and VH within an integrated framework. This refined classification method is more precise than traditional single-trust measurement approaches and helps reveal how various types of trust influence VH and vaccination behavior. The findings indicate that different types of social trust exert varying effects on VH. After adjusting for all covariates, the results suggest that trust in doctors and the government exerts a more significant influence on respondents’ VH. This may stem from China’s unique cultural context, where people traditionally place greater trust in the government and medical professionals. During COVID-19, China made great efforts to control the spread of the outbreak. These outbreak prevention and control measures included conducting mass nucleic acid testing, requiring mask-wearing in public places, and organizing medical experts to provide epidemic briefings. They were well implemented in China precisely because of the high level of trust in the government and doctors while protecting people’s health at the same time. As shown by a qualitative study conducted in China, access to healthcare workers’ professional advice played a crucial role in individuals’ vaccination decisions. All 92 participants in the qualitative interviews recognized doctors’ advice as “professional,” “constructive,” “widely accepted,” “decisive,” and “official” (59). Additionally, the findings of this study also indicate that increased levels of internet trust can reduce the likelihood of VH. This result differs from conclusions drawn in some existing studies, which suggest that misinformation about vaccines circulating online negatively impact audiences, diminishing their confidence in vaccines and increasing VH (60, 61). This study suggests that the reasons for these differing outcomes may lie in the unique characteristics of China’s internet trust mechanisms, with factors such as the degree of government control, platform compliance, and information transparency all play significant roles. In China, the internet environment is strictly regulated and censored. Internet companies must comply with government laws and regulations, including those related to data privacy, security, and content review. These companies are often required to cooperate with government monitoring and inspection of user data, which impacts public trust in internet platforms. Secondly, Chinese internet users rely more heavily on official and platform guidance in information dissemination. Trust mechanisms are often based on government and platform compliance and direction, rather than solely on users’ independent judgment. This differs from internet cultures in many Western countries, which emphasize freedom of speech and decentralized trust mechanisms. Consequently, even when misinformation circulates on social media, the public generally trusts information released by the government and state-owned media. In the Chinese context, internet trust primarily reflects confidence in official vaccine information, helping to reduce VH. However, the measurement of internet trust in this study may not fully capture the multidimensional nature. The complexity of internet trust likely includes multiple dimensions, such as judgment of information sources, assessment of information accuracy, and trust in information dissemination platforms. Operationalizing this concept with a single question may not comprehensively capture its essence. Future research could consider using more refined scales to measure internet trust. Finally, regarding the relationship between generalized trust and VH, our findings reveal a significant negative correlation between generalized trust and VH. This indicates that enhancing generalized trust can help reduce the likelihood of hesitancy. This result differs from existing research, which has focused primarily on the impact of various specific types of trust on VH (62, 63). The findings of this study indicate that, in addition to various specific forms of trust, a general sense of trust and broad social trust at the societal level also contribute to reducing VH. Therefore, improving risk management for infectious diseases and reducing VH requires not only enhancing medical conditions but also prioritizing the establishment of interpersonal trust. However, the measurement of generalized trust in this study may oversimplify the complexity of trust, as generalized trust encompasses multiple dimensions involving social trust, personal trust, and trust in institutions. While the measurement questions employed in this study provide an effective summary metric, they cannot fully represent the multidimensional structure of generalized trust. Future research could use multiple indicators to achieve a more comprehensive measurement.

Structural equation modeling analyses revealed the psychological mechanisms underlying the association between trust and VH by quantifying the mediating role of the “3C” psychological antecedents of vaccination. Previous studies have revealed the mediating role of the psychological antecedents of vaccination between information perception and VH, suggesting that the negative effects of misinformation on VH may be further enhanced by the psychological and emotional responses expressed in the psychological antecedents of vaccination (64). In contrast, this study indicated that the association between trust and VH was strengthened by the psychological and emotional responses of self-confidence, complacency, and collective responsibility in the “3C” psychological antecedents of vaccination. This research further enhances our understanding of the relationship between trust and VH. It reveals the multidimensional nature of trust and demonstrates how different types of trust indirectly influence the underlying mechanisms of VH. This provides a more multi-layered perspective for understanding VH and helps address gaps in existing studies by integrating trust types with mediating mechanisms. According to the knowledge-attitude-practice theory of health behavior, acquired positive health information can effectively increase an individual’s awareness of the benefits of health behaviors and enhance the individual’s behavioral motivation, thus promoting the individual’s performance of positive behaviors (65, 66). During the epidemic, various types of trust obtained from the outside world can be regarded as a kind of positive health improvement information (e.g., wearing masks advocated by the government, professional advice given by doctors, or real and effective life skills shared on the Internet, etc.). Increased acceptance of such information not only enhances individuals’ understanding of vaccination effectiveness and reduces misunderstandings about vaccine information, but also promotes compliance with collective vaccination initiatives, thereby reducing vaccination barriers and encouraging vaccine uptake.

In addition, the results firstly showed that respondents were vaccinated with COVID-19 mainly through community organization/notification, which proved the importance of community participation in addressing public health challenges and its role in promoting healthy behaviors and preventing infectious diseases. This aligns with another Chinese study showing a positive correlation between community involvement and vaccination rates, where individuals receiving community notifications were more likely to get both COVID-19 and influenza vaccines (51). Secondly, the results also showed that Chinese respondents also experienced delays in the vaccination process, while the degree of delay was negatively correlated with the level of trust. A similar pattern was found in a previous COVID-19 study conducted in Mexico (67).

This study is relevant to the field of infectious diseases in several ways. First, this study used representative survey data from China to demonstrate that trust among social factors is a key factor influencing vaccination. Current research on factors influencing VH focuses on factors such as the vaccine itself, while fewer studies have focused on trust. However, in the absence of trust, the public is less likely to cooperate with government policies adopted to prevent disease and promote vaccination. Especially during times of outbreak prevention and control, trust is an essential component of effective healthcare delivery (68), and the public’s cooperation is critical to effectively controlling the development of an outbreak. Second, there is no research on the mechanisms between the two variables, so how various types of trust VH is unclear, and the mechanisms of their influence have been overlooked. This study not only analyzes how trust affects VH at both theoretical and empirical levels but further validates this mechanism using representative data, which provides a new academic contribution to hesitancy-related research. Finally, this study reveals the impact of different types of trust on VH. Most previous studies on trust and VH focused on only one type of trust. However, this study reflect the entire structure of the relationship between trust and VH. This study provides empirical evidence that different types of trust have different effects on VH, suggesting that several types of trust should be treated separately in studies of VH interventions.

## 6 Policy recommendations

Based on the above discussion, we propose the following policy recommendations. First, enhancing trust levels as an intervention may help prevent and mitigate VH, particularly in today’s society, which is facing numerous infectious disease threats. Research shows that generalized trust significantly reduces VH, indicating that such trust lowers individuals’ perceived risk of the disease. Individuals more likely to trust others unconditionally also tend to have more optimistic views about perceived risks. Public trust can be built through dedicated initiatives, such as regularly organizing open forums and symposiums. These events should invite experts, government officials, and community representatives to engage in face-to-face dialog with the public, addressing concerns about vaccines, public health policies, and related issues. Second, as policymakers, governments play a crucial role in epidemic prevention and control. To improve public compliance with government policies during emergencies like epidemics—such as encouraging mask-wearing or social distancing during COVID-19—it is essential to strengthen public trust in the government. During outbreaks, governments should prioritize disseminating authoritative information, promptly communicating epidemic policies, and maintaining open dialog with the public. This is because the performance of public services is the most critical factor citizens use to judge whether a government is trustworthy, and improving public service delivery is an effective way to build public trust. For instance, establishing an official pandemic information website and disseminating policies and health advisories through social media platforms are advisable. Moreover, enhancing crisis communication capabilities among government officials is crucial. Training government officials and public health experts to improve their ability to communicate with the public during pandemics—particularly in explaining complex pandemic policies using plain language—is essential. Finally, implementing a “Government Trust Survey” mechanism to regularly gauge public trust in the government and adjust policy communication strategies based on survey results is recommended. Third, physicians can play a pivotal role in reducing vaccine hesitancy. By leveraging their expertise, doctors can communicate the value of vaccination to the public and increase awareness of vaccine safety and efficacy. However, research on VH in China indicates that physicians are not highly proactive in recommending vaccines to patients during clinical practice, and their potential to mitigate VH remains underused (69). Therefore, further efforts to reduce VH should focus on enhancing the professional role of physicians. First, physician training programs should be established. Systematic training plans should be developed to conduct regular vaccination training sessions for doctors, covering vaccine safety, efficacy, and strategies for addressing patient concerns. Second, a “Physician Vaccination Recommendation” initiative should be promoted, encouraging doctors to proactively recommend vaccines during routine medical visits and provide personalized health advice. Incentives, such as rewards or recognition for doctors who actively promote vaccines, could be established. Finally, institutional support for healthcare providers must be strengthened. Healthcare institutions should allocate more resources and time to enable physicians to effectively communicate vaccine information to patients during routine consultations. Fourth, while misinformation about vaccines online impacts public vaccination rates, the internet undeniably provides a more convenient channel for the public to access health information. This positive role is particularly evident in China’s unique online trust environment. Individual attitudes toward information shape health behaviors, and online health information seeking positively influences personal health actions. To maximize the internet’s role in disseminating scientific vaccine information, first, establish fact-checking partnerships with social media platforms. Governments and public health organizations should collaborate with major platforms to create fact-checking mechanisms for timely review and removal of false vaccine information. Second, promote online health education initiatives use social media platforms and websites to implement systematic vaccine education programs, providing the public with verified scientific data and knowledge to help them distinguish between accurate and misleading information. Finally, strengthen public health literacy efforts. Regularly disseminate vaccine science through social media, television, radio, and other channels to address public concerns, particularly offering detailed explanations regarding the safety and efficacy of COVID-19 vaccines and other immunizations. Fifth, enhance community engagement in vaccination efforts by strengthening vaccine education and promotion at the community level to increase societal acceptance. This can be achieved through enhanced collaboration between community organizations and health institutions, utilizing community platforms to conduct comprehensive vaccine education and outreach campaigns to ensure accurate information dissemination. Additionally, training community workers on vaccine knowledge can empower them to explain the necessity and safety of vaccination to residents. Additionally, organize community vaccination days and establish temporary vaccination sites within neighborhoods to facilitate convenient access for residents.

## 7 Strengths and limitations

To our knowledge, this is the first study to use a large-scale Chinese micro-level survey database to examine the relationship between trust and VH, as well as the mediating role of the “3C” psychological antecedents in this relationship. By distinguishing different types of trust, this study enables a more precise analysis of how trust influences VH. Furthermore, by linking various trust types to mediating variables, we gain deeper insights into the distinct pathways through which trust affects vaccination decisions, thereby revealing the underlying mechanisms through which trust indirectly influences VH. While this research offers valuable insights into the relationship between trust and VH, several limitations warrant further exploration in subsequent studies. First, the VH measured in this study is based on self-reporting, potentially introducing recall bias. Second, while our findings demonstrate an association between trust and VH, the cross-sectional design precludes establishing causality. This design limitation prevents conclusive evidence of trust’s causal effect on VH, and further causal inferences require validation using longitudinal data. Third, other factors and mediating pathways influencing VH may exist. These include health insurance status and chronic disease conditions, among others. Additionally, alternative mediating pathways—such as convenience—could be involved. Due to the lack of suitable proxy variables, these factors were not included into the current analysis. Future studies with more comprehensive data should explore their influence and mediating roles. Fourth, due to data availability constraints, the dependent variable was defined as a binary outcome of “vaccinated” versus “unvaccinated.” However, individuals who remain unvaccinated may not necessarily do so due to hesitancy or refusal, but rather due to other factors such as vaccine accessibility issues. We acknowledge that the current dependent variable definition may conflate “VH” with “vaccine accessibility.” However, the existing dataset does not include detailed self-reported hesitancy data (e.g., “delayed/hesitant but vaccinated” versus “refused vaccination”), preventing us from using these as primary dependent variables in sensitivity analyses. Future research could use more granular self-reported data to further distinguish between “vaccine-hesitant” and “vaccine-refusing” groups. This would better separate accessibility issues from hesitancy and enable a more precise analysis of trust’s impact on VH, thereby providing more accurate conclusions and revealing trust’s true influence on VH. Fifth, due to data availability constraints, generalized trust in this study was measured using a single question, which may oversimplify the complexity of trust. Future research could employ multiple indicators to measure generalized trust more comprehensively. Additionally, internet trust was operationalized as perceiving online information as beneficial, an approach that may inadequately capture the essence of “trust” and risks conflating information exposure with credibility. Future research may consider operationalizing internet trust through more granular scales, such as using multiple questions to separately assess respondents’ trust in various information sources (e.g., social media, news websites). This approach would facilitate a more comprehensive understanding of trust’s influence on behaviors like VH.

## 8 Conclusion

This study provides an in-depth investigation of the relationship between trust and VH, revealing underlying psychological mechanisms. Through structural equation modeling, multiple factors influencing VH were identified and the connection between levels of VH and trust was quantified. Trust represents a unique form of social capital. During epidemics, careful attention should be paid to both the interrelationships among different types of institutional trust and their connections with vaccine attitudes, in order to maximize trust’s potential in reducing VH.

## Data Availability

This study was based on a publicly available database. Dataset/questionnaire/interview used in your study has previously been published elsewhere, those informations are available through the China Survey and Data Center at Renmin University of China. The datasets generated and/or analyzed during the current study can be found at: http://cgss.ruc.edu.cn/.
