# Correction: Understanding vaccine hesitancy through the lens of trust and the 3C model: evidence from Chinese General Social Survey 2021

**DOI:** 10.3389/fpubh.2025.1730651

**Published:** 2025-11-11

**Authors:** Bo Dong, Hengxuan Xu, Yuantao Qi, Yifan Li

**Affiliations:** 1 School of Public Health, Zhejiang Chinese Medicine University, Hangzhou, Zhejiang, China; 2 Zhongnan Hospital of Wuhan University, Wuhan, Hubei, China; 3 Shandong Cancer Hospital and Institute, Shandong First Medical University and Shandong Academy of Medical Sciences, Jinan, Shandong, China

**Keywords:** trust, vaccine hesitancy, COVID-19, psychological antecedent, China

There was a mistake in affiliation 1 as published. Affiliation “School of Public Health, Zhejiang Chinese Medicine University, Hangzhou, Zhejiang, China” was erroneously given as “School Public Health, Zhejiang Chinese Medicine University, Hangzhou, Zhejiang, China”.

There was a mistake in **Figure 6(e)** as published. The corrected Figure 6(e) appears below.

**Figure 6:**
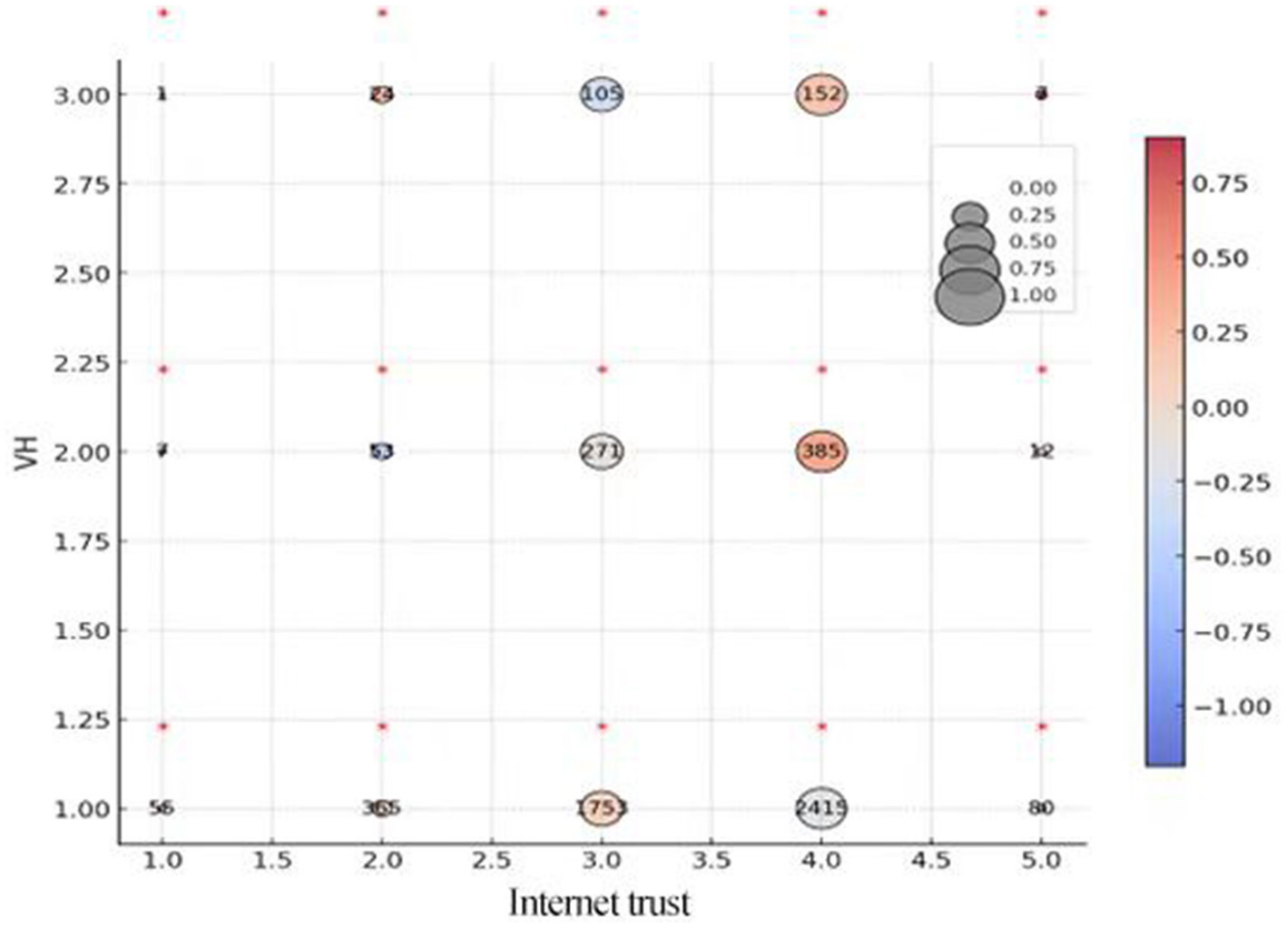
Level of vaccine hesitation and type of trust. This figure shows a bar graph and 5 contingency tables that follow the scale located in the upper right section of the figure. **(a)** Respondents' approach to COVID-19 vaccines. **(b)** Distribution of vaccine hesitancy on Generalized trust. **(c)** Distribution of vaccine hesitancy on government trust. **(d)** Distribution of vaccine hesitancy on doctor trust. **(e)** Distribution of vaccine hesitancy on internet trust. The numbers inside the circles show the absolute frequency, the size of the circle indicates the proportion relative to the level of VH, and the color of the circle shows the value of the standardized residuals of the Chi-squared for each cell. The standardized residuals whose value is higher than 2 or lower than−2 are considered as significant differences and are highlighted with an asterisk above the circle.

There was a mistake in the caption of **Figure 6** as published. The corrected caption of Figure 6 appears below.

The original version of this article has been updated.

