# Supplementary material for: Understanding vaccine hesitancy through the lens of trust and the 3C model: evidence from Chinese General Social Survey 2021
Source: Front Public Health. 2025 Oct 1;13:1671457. doi: 10.3389/fpubh.2025.1671457 (PMC12521134; doi:10.3389/fpubh.2025.1671457)
Supplement: Supplementary file 1 [file Data_Sheet_1.doc]

**Appendix**

**Table 1** Selection of variable measurement questions in CGSS (2021)

| **Variables** | | **Measurement problems** |
| --- | --- | --- |
| Independent variables | VH | Currently, have you been vaccinated against COVID-19? |
| Dependent variables | Generalized trust | Overall, do you agree that the vast majority of people can be trusted in this society |
|  | Government trust | How has China's response to the COVID-19 epidemic changed your confidence in the government? |
|  | Doctor trust | Do you think Chinese doctors can be trusted? |
|  | Internet trust | Do you agree that information on the Internet has had a positive impact on your health behaviors in the past 12 months? |
| Intermediate variables | Confidence | Overall, the benefits of vaccination outweigh the disadvantages |
|  |  | Immunity through vaccination is better than illness. |
|  | Complacent | China's epidemic is well under control, no need for vaccination |
|  |  | Everyone else has been inoculated. There's no need to inoculate yourself. |
|  |  | Worried that after vaccination, prevention is not effective |
|  |  | Fear of adverse health effects after vaccination |
|  |  | Fear of ineffective government management of vaccines (fake vaccines, failed vaccines, etc.) |
|  |  | Want to get vaccinated in other countries |
|  | Collective responsibility | “If I get the COVID-19 coronavirus, it's my own fault”, do you agree? |
|  |  | “If someone gets infected with the COVID-19 coronavirus, it's their own fault”, do you agree? |
| Control variables | Gender | What is your date of birth? |
|  | Age | Please select your gender |
|  | Income | What is your total annual household income? |
|  | Religious Belief | What are your religious beliefs? |
|  | Education | What is your current highest level of education? |
|  | Work | What is your current work history and status? |
|  | Health | How do you feel about your current health? |
|  | Marriage | What is your marital status? |
|  | Household Registration | What is your current household registration status? |
|  | Ethnicity | What is your ethnicity? |
|  | Region | Province of the respondent |

**Table 2**  Variable Definitions

| **Variables** | | **Definitions** |
| --- | --- | --- |
| Independent variables | VH | No vaccination = 0 |
|  |  | Vaccination =1 |
| Dependent variables | Generalized trust | Strongly disagree = 1 |
|  |  | Disagree = 2 |
|  |  | Can't say agree or disagree =3 |
|  |  | Agree = 4 |
|  |  | Strongly agree = 5 |
|  | Government trust | Much lower = 1 |
|  |  | Lowered a bit = 2 |
|  |  | Basically unchanged =3 |
|  |  | Grew a little = 4 |
|  |  | It's grown a lot =5 |
|  | Doctor trust | Strongly disagree = 1 |
|  |  | Disagree = 2 |
|  |  | Can't say agree or disagree =3 |
|  |  | Agree = 4 |
|  |  | Strongly agree = 5 |
|  | Internet trust | Strongly disagree = 1 |
|  |  | Disagree = 2 |
|  |  | Can't say agree or disagree =3 |
|  |  | Agree = 4 |
|  |  | Strongly agree = 5 |
| Intermediate variables | Confidence | Strongly disagree |
|  |  | Disagree |
|  |  | Can't say agree or disagree |
|  |  | Agree |
|  |  | Strongly agree |
|  |  | Take the total score for each dimension of the two questions |
|  | Complacent | Select one of the different dimensions to be assigned a point, and stack them cumulatively. |
|  | Collective responsibility | Disagree |
|  |  | Disagree more |
|  |  | Comparative agreement |
|  |  | Agree |
|  |  | Take the total score for each dimension of the two questions |
| Control variables | Gender | ≤29=1；30-59=2；≥60=3 |
|  | Age | Male = 1; Female = 2 |
|  | Income | <20000=1 |
|  |  | 20000-=2 |
|  |  | 50000-=3 |
|  |  | 100000-=5 |
|  |  | 200000-=5 |
|  | Religious Belief | No religion = 0; religion = 1 |
|  | Education | Less than high school = 1; high school = 2; college and beyond = 3 |
|  | Work | Without work = 1; with work 2 |
|  | Health | Self-rated health: unhealthy = 1; mostly healthy = 2; healthy = 3 |
|  | Marriage | Unmarried = 1; Married = 2 |
|  | Household Registration | Rural = 1; Urban = 2 |
|  | Ethnicity | Han =1; Ethnic minority =2 |
|  | Region | West = 1; Central = 2; East = 3 |
